# Evaluating the Anti-cancer Efficacy of a Synthetic Curcumin Analog on Human Melanoma Cells and Its Interaction with Standard Chemotherapeutics

**DOI:** 10.3390/molecules24132483

**Published:** 2019-07-06

**Authors:** Krishan Parashar, Siddhartha Sood, Ali Mehaidli, Colin Curran, Caleb Vegh, Christopher Nguyen, Christopher Pignanelli, Jianzhang Wu, Guang Liang, Yi Wang, Siyaram Pandey

**Affiliations:** 1 Department of Chemistry and Biochemistry, University of Windsor, 401 Sunset Avenue, Windsor, ON N9B 3P4, Canada; 2 Chemical Biology Research Center, School of Pharmaceutical Sciences, Whenzhou Medical University, University Town, Chashan, Wenzhou 325035, China

**Keywords:** melanoma, curcumin analog, apoptosis, oxidative stress, drug–drug interaction, tamoxifen, taxol, cisplatin

## Abstract

Melanoma is the leading cause of skin-cancer related deaths in North America. Metastatic melanoma is difficult to treat and chemotherapies have limited success. Furthermore, chemotherapies lead to toxic side effects due to nonselective targeting of normal cells. Curcumin is a natural product of *Curcuma longa* (turmeric) and has been shown to possess anti-cancer activity. However, due to its poor bioavailability and stability, natural curcumin is not an effective cancer treatment. We tested synthetic analogs of curcumin that are more stable. One of these derivatives, Compound A, has shown significant anti-cancer efficacy in colon, leukemia, and triple-negative inflammatory breast cancer cells. However, the effects of Compound A against melanoma cells have not been studied before. In this study, for the first time, we demonstrated the efficacy of Compound A for the selective induction of apoptosis in melanoma cells and its interaction with tamoxifen, taxol, and cisplatin. We found that Compound A induced apoptosis selectively in human melanoma cells by increasing oxidative stress. The anti-cancer activity of Compound A was enhanced when combined with tamoxifen and the combination treatment did not result in significant toxicity to noncancerous cells. Additionally, Compound A did not interact negatively with the anti-cancer activity of taxol and cisplatin. These results indicate that Compound A could be developed as a selective and effective melanoma treatment either alone or in combination with other non-toxic agents like tamoxifen.

## 1. Introduction

Melanoma is an aggressive malignancy that emerges from the uncontrolled division of melanocytes and contributes to the larger part of skin cancer deaths [1]. Global incidence of melanoma has increased significantly, with rates rising in Europe and North America [2,3,4]. Fortunately, melanoma is easily treatable by surgical removal if detected early [5]. Deep melanoma tumors, however, tend to metastasize to lymph nodes and spread to other parts of the body [5,6]. Hence, at advanced stages surgery is not adequate and treatment becomes more difficult. Further treatment options include chemotherapy, radiation, and immunotherapy [7]. Melanoma is known to be highly resistant, thus limiting the effectiveness of these therapies [7,8]. Most chemotherapies are genotoxic or target cytoskeleton structures in cancer cells to induce cell death. However, this targeting is nonselective as it kills normal cells, which leads to severe toxicity.

Recent research has focused on targeting mitochondria, oxidative stress, and metabolic vulnerabilities to induce cancer cell death selectively [9]. Cancer cells shift their metabolism from oxidative phosphorylation to glycolysis, which reduces mitochondrial permeabilization and promotes mitochondrial stability [10,11,12,13]. The shift to glycolysis and the up-regulation of anti-apoptotic proteins, enables cancer cells to resist apoptosis [11,12,13]. Induction of cancer cell death selectively can occur through targeting the unique characteristics of cancerous cells. For example, analogs of the natural compound pancratistatin (PST) were able to induce apoptosis selectively in cancer cells through disrupting the mitochondrial membrane potential and the release of apoptogenic factors [14,15]. Cancer cells also display higher basal levels of reactive oxygen species (ROS), which promotes tumor proliferation and progression [16,17]. However, excessive ROS levels lead to damage of important biomolecules including DNA and protein, thus resulting in cell death [18]. Cancer cells depend on up-regulated expression of antioxidant enzymes to survive. Therefore, external sources of ROS or agents that stimulate oxidative stress may target cancer cells selectively [16]. For example, the natural compound piperlongumine induces cell death selectively through targeting the oxidative pathway [14]. An enhanced cytotoxic effect was observed when piperlongumine was combined with a pancratistatin analog, an activator of the intrinsic pathway of apoptosis through mitochondrial targeting [15].

Curcumin is a natural compound isolated from the *Curcuma longa* plant and has been shown to inhibit cancer growth and induce apoptosis in cancer cells [19,20]. Curcumin is pleiotropic and affects the activity of signaling molecules in a variety of pathways including inflammation [21]. Interestingly, curcumin has been shown to induce cell death through increasing ROS [20,22,23]. Due to poor bioavailability and stability, curcumin is not effective in vivo models and therefore could not advance to clinical success [24]. However, synthetic analogs of natural curcumin could have increased chemical stability and bioavailability. Therefore, these molecules should have the potential to be developed as cancer-selective drugs. Furthermore, a more potent analog could be synthesized that may have very high anti-cancer activity at low concentrations.

We synthesized several novel analogs of curcumin and screened them on various cancer cell lines [24]. Previously, we have demonstrated that two analogs, Compounds A and I, were the most effective in inducing apoptosis selectively in different cancer cell lines including triple-negative breast and p53-negative colorectal cancer cells [24]. Furthermore, these analogs induced cell death at lower doses compared to natural curcumin and the induction of apoptosis was driven by oxidative stress selectively in cancer cells. Compound A was also found to be effective in inhibiting human tumor growth xenografted in nude mice when administered intraperitoneally. This suggested that Compound A is biostable as well as bioavailable. Additionally, Compound A was shown to be well tolerated in mice. However, the anti-cancer activity of Compound A and other analogs of curcumin had yet to be studied in human melanoma cells. The interactions of these compounds with standard chemotherapies have also not been investigated.

Tamoxifen (TAM) is a non-genotoxic drug used to treat and prevent estrogen receptor (ER) positive breast cancer [25]. Though tamoxifen functions as an ER antagonist, it has also been shown to target and disrupt the mitochondria [25,26]. Previous work demonstrated that tamoxifen sensitized cancer cell mitochondria, thereby enhancing the anti-cancer efficacy of PST in ER negative breast cancer, and melanoma cells [27,28]. In a previous study, natural curcumin was combined with tamoxifen, which resulted in a synergistic induction of cell death selective to melanoma cells [29]. Conversely, this combination treatment did not result in significant cell death in noncancerous cells. Cell death was attributed to apoptosis as well as autophagy, a pro-survival or pro-death process, which occurs in response to stress [30,31]. Given that Compound A is more effective than natural curcumin, it is imperative to also investigate the interaction of Compound A with tamoxifen on human melanoma cells.

The objective of this study was to investigate the efficacy of novel synthetic curcumin analogs against human melanoma cells and demonstrate the possible mechanism of induction of apoptosis. We determined the effect of combining Compound A with tamoxifen in melanoma cells. We also investigated the drug–drug interactions of Compound A in combination with the standard chemotherapeutics taxol and cisplatin. Through screening the analogs on melanoma cells, Compound A was determined to be the most effective and selective in reducing cell viability. We have observed the selective induction of apoptosis by Compound A in two different melanoma cell lines. Furthermore, the effective doses of Compound A were well tolerated in normal human fibroblasts. Investigation into the mechanism revealed that cell death was triggered through induction of oxidative stress. The combination treatment of low doses of Compound A and tamoxifen resulted in an enhancement of apoptosis in human melanoma cells. Lastly, Compound A did not interfere with the anti-cancer activity of taxol and cisplatin. In conclusion, in this paper we demonstrate for the first time the anti-cancer activity of Compound A against human melanoma cells. These results may lead to the development of a non-toxic treatment that induces apoptosis selectively in melanoma cells through targeting oxidative vulnerabilities. Additionally, we demonstrate that Compound A has the potential to be developed as an adjuvant with tamoxifen and as a safer and effective melanoma treatment.

## 2. Results

### 2.1. Compounds A and I Induce Cell Death Effectively and Selectively in Human Melanoma Cells

**Figure 1:**
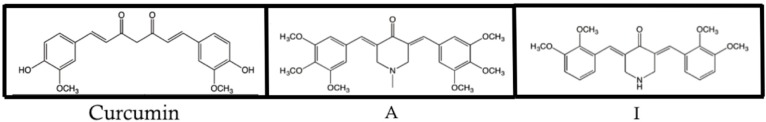
Structures of curcumin and analogs A and I. Previously published in Pignanelli et al. [24].

**Figure 2:**
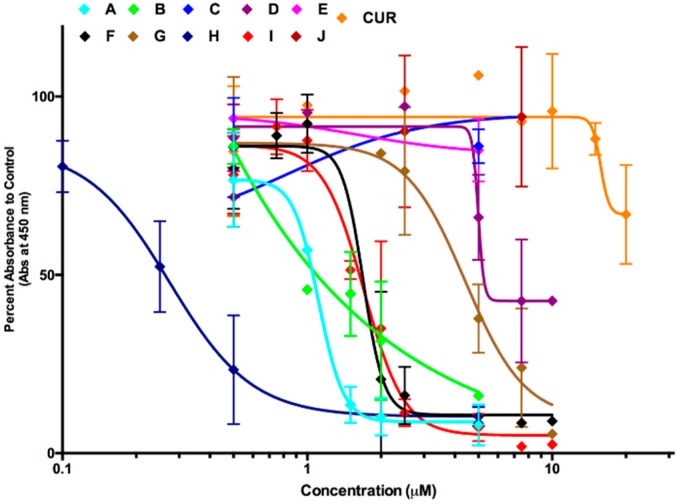
Effect of various curcumin analogs on cell viability of A375 melanoma cells. A375 melanoma cells were treated with natural curcumin (CUR) and curcumin analogs (A-J) for 48 h. The WST-1 assay was performed as described in the materials and methods section. Values are expressed as a mean ± SD from at least three independent experiments. The *x*-axis represents concentration (μM) and the *y*-axis represents absorbance (% of control). Graph obtained using the log (inhibitor) vs. response—variable slope (four parameters) curve on GraphPrism6.

**Figure 3:**
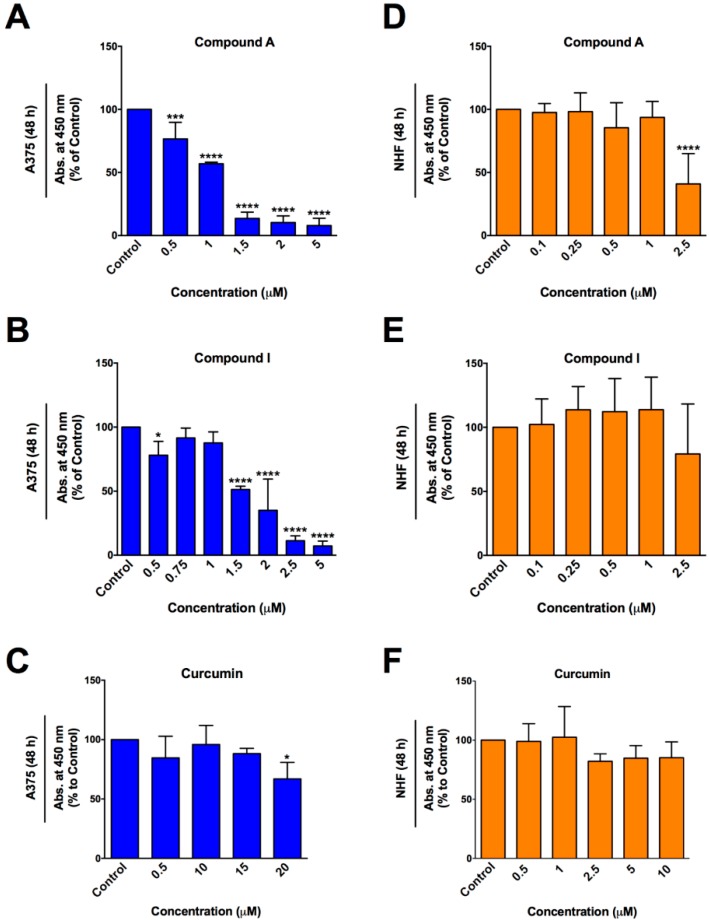
Compounds A and I selectively reduce cell viability in human melanoma cells. (**A–C**) A375 melanoma cells and (**D–F**) noncancerous normal human fibroblasts (NHF) were treated with Compound A, Compound I, and curcumin for 48 h. The WST-1 assay was performed as described in the materials and methods section. A375 cell viability data from Figure 1 were re-plotted as bar graphs to produce Figure 3 (A–C). Values are expressed as a mean ± SD from at least three independent experiments. The *x*-axis represents concentration (μM) and the *y*-axis represents absorbance (% of control).

The WST-1 colorimetric assay was utilized 48 h post treatment to determine the anti-cancer efficacy of curcumin (Figure 1) and synthetic analogs of curcumin (A-J), on A375 melanoma cells (Figure 2). Overall these analogs were more effective in reducing cell viability in A375 cells than natural curcumin. Natural curcumin was almost ineffective in reducing the viability of A375 cells even up to 20 μM (Figure 3C). However, various synthetic analogs were effective in reducing the viability of these cells at lower doses. The viability data for Compound A, I, and curcumin (for structures see Figure 1) were re-plotted as bar graphs for better comparison (Figure 3A–C). Compounds A and I were the most effective in reducing cell viability as demonstrated by lower IC_50_ values (approximately 1 and 2 μM, respectively; Figure 3A,B). The effective doses of Compounds A and I were tested on noncancerous normal human fibroblasts (NHF) and were well tolerated (Figure 3D,E). Similarly, natural curcumin did not reduce cell viability in NHF cells at different doses as indicated in Figure 3F. These results indicate selective toxicity of Compounds A and I to melanoma cells. It is important to note that Compound H was very effective in killing cancer cells at lower doses but was not taken for further study due to similar toxicity to normal cells, as determined previously [24]. Thus, Compounds A and I were chosen for further investigation with regards to the mode of cell death and mechanism.

### 2.2. Compounds A and I Induce Apoptosis Selectively in Human Melanoma Cells

**Figure 4:**
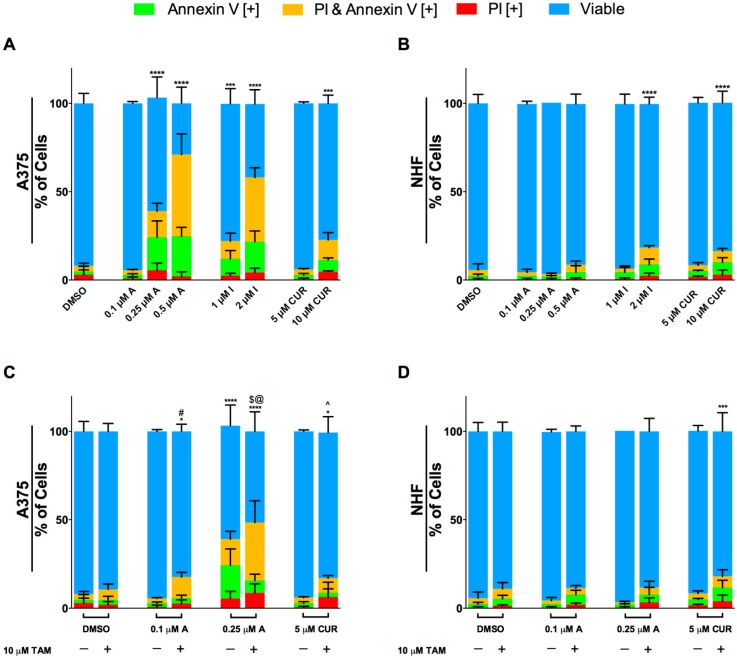

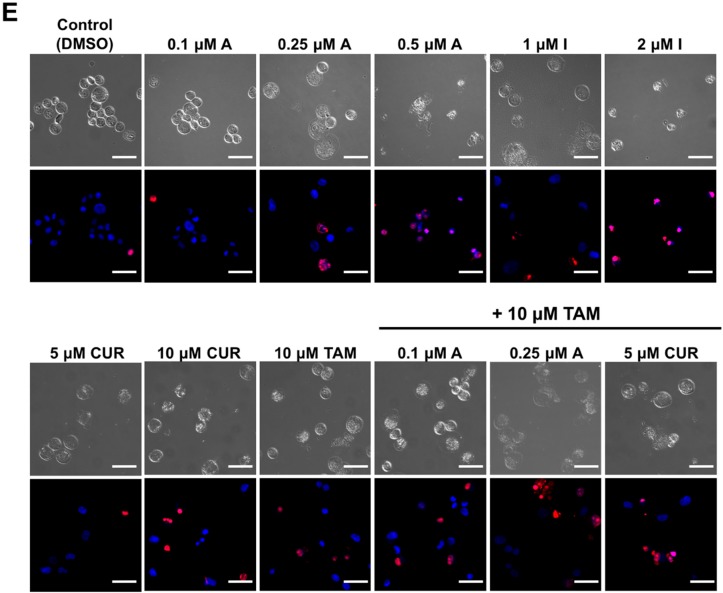
Compounds A and I induce apoptosis selectively and Compound A interacts positively with tamoxifen. (**A**) A375 melanoma cells and (**B**) noncancerous normal human fibroblasts (NHF) were treated with the indicated compounds for 48 h then stained with Annexin V and PI to quantify apoptosis, as described in the materials and methods section. Image based cytometry was utilized to assess apoptosis. The *y*-axis represents the percent of cells positive for Annexin V (green), PI (red), Annexin V and PI (orange), or cells negative for Annexin V and PI (blue). Values are expressed as mean ± SD from three independent experiments. (**C**) A375 melanoma cells and (**D**) NHF cells were treated with Compound A and curcumin alone and in combination with tamoxifen for 48 h. Please note, the data of Compound A and curcumin alone shown in Figure 4A,B were used again in Figure 4C,D, respectively along with the combination treatments for direct comparison. *p < 0.05 vs. DMSO control (comparison of viable cells only); **p < 0.01 vs. DMSO control (comparison of viable cells only); ***p < 0.001 vs. DMSO control (comparison of viable cells only); ****p < 0.0001 vs. DMSO control (comparison of viable cells only); #*p* < 0.05 vs. 0.1 μM Compound A alone (comparison of viable cells only); $*p* < 0.05 vs. 0.25 μM Compound A alone (comparison of viable cells only); ^*p* < 0.05 vs. 5 μM CUR alone (comparison of viable cells only); @p < 0.05 vs. tamoxifen treatment alone (comparison of viable cells only). (**E**) A375 micrographs at 48 h. Top: Bright field images at 400× magnification. Bottom: Fluorescent images stained with PI (red) and Hoechst (blue). Scale bar = 100 μm. Micrographs are representative of three independent experiments. A = Compound A, I = Compound I, CUR = Curcumin, TAM = Tamoxifen.

The Annexin V binding assay and propidium iodide (PI) staining was utilized to distinguish early and late stage apoptosis, respectively. A375 (Figure 4A) and NHF (Figure 4B) cells were treated for 48 h with Compound A, Compound I, and curcumin. Compounds A and I induced apoptosis effectively in A375 melanoma cells at significantly lower doses compared to natural curcumin (Figure 4A). As shown in Figure 4A, Compound A induced significant apoptosis at 0.25 μM and 0.5 μM doses whereas limited induction of apoptosis was observed by natural curcumin only at 10 μM dose. Interestingly, the doses of Compound A that were greatly toxic to melanoma cells did not induce apoptosis significantly in noncancerous normal human fibroblasts 48 h following treatment (Figure 4B). However, significant apoptotic induction was observed at higher doses of Compound I (2 μM) and curcumin (10 μM). These results were supported by cellular and nuclear morphology following staining of A375 cells with Hoechst and propidium iodide (Figure 4E). The images revealed morphological changes indicative of apoptosis such as cell shrinkage, nuclear condensation, and PI positive signals (indicating permeability) in cancer cells.

### 2.3. Enhancement of the Anti-cancer Activity of Compound A in Combination with Tamoxifen

Tamoxifen as such does not have strong anti-cancer activity against melanoma cells. Previously it has been reported that tamoxifen in combination with curcumin demostrated sufficient cytotoxicity against melanoma cells [29]. We investigated whether the synthetic curcumin derivative, Compound A would show a similar enhancement wth tamoxifen. A375 cells were treated with Compound A and tamoxifen alone or in combination for 48 h and apoptosis was monitored using Annexin V binding assay and propidium iodide staining. As shown in Figure 4C, there was a clear enhancement of cell death inducing activity of Compound A by tamoxifen particularly at low doses of Compound A (0.1 μM and 0.25 μM). These results were supported by fluorescent micrographs of A375 cells treated with the indicated compounds for 48 h followed by staining with Hoechst and propidium iodide (Figure 4E). On the other hand, the same combination did not show significant toxicity to normal human fibroblasts (Figure 4D).

### 2.4. Interaction of Compound A with Taxol and Cisplatin in Two Melanoma Cell Lines

**Figure 5:**
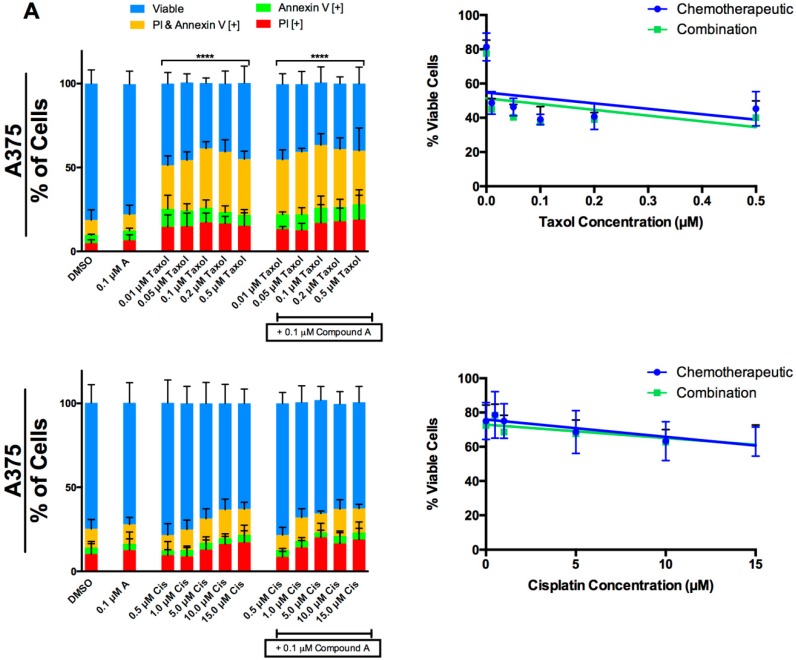

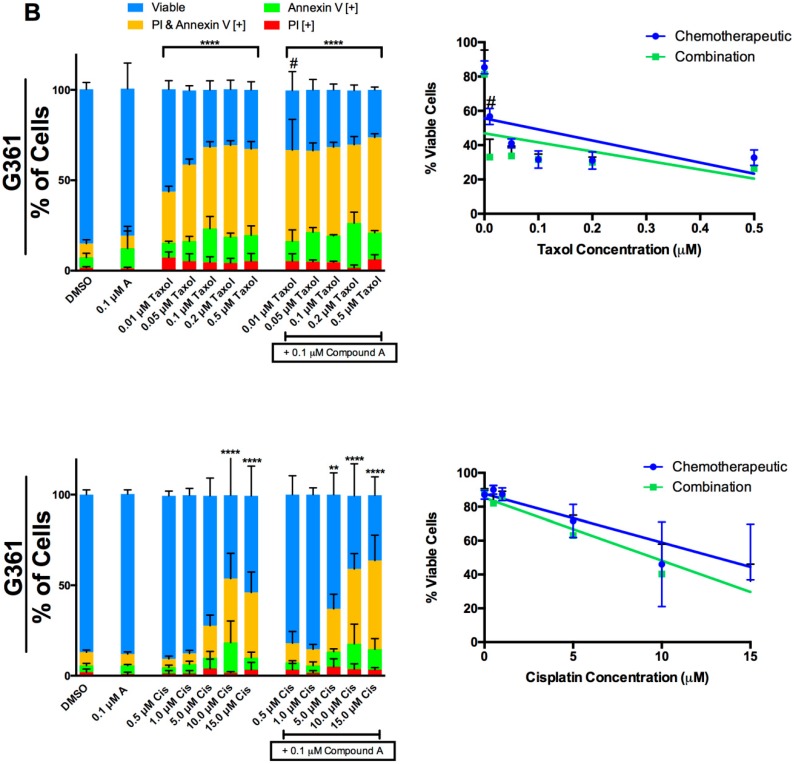
The interaction of Compound A with taxol and cisplatin in two melanoma cell lines. (**A**) A375 and (**B**) G361 melanoma cells were treated with a range of taxol (top panel) and cisplatin concentrations (bottom panel) alone and in combination with a sub lethal dose of Compound A (0.1 μM). Following 48 h of treatment the cells were stained with Annexin V and PI to distinguish early apoptosis and late stage apoptosis, respectively. Image based cytometry was utilized to assess apoptosis. The *y*-axis represents the percent of cells positive for Annexin V (green), PI (red), Annexin V and PI (yellow), or negative for both Annexin V and PI (blue). All graphical values are expressed as a mean ± SD from three independent experiments. A line graph was constructed using viability values for individual chemotherapeutics in the absence and presence of Compound A (combination). **p < 0.01 vs. DMSO control (comparison of viable cells only); ****p < 0.0001 vs. DMSO control (comparison of viable cells only); #*p* < 0.0001 vs. 0.01 μM taxol alone (comparison of viable cells only). Cis = Cisplatin.

We investigated the effect of Compound A on the cell-death-inducing activity of taxol and cisplatin on human melanoma cells. A375 (Figure 5A) and G361 (Figure 5B) cells were treated with a range of taxol and cisplatin doses in the presence or absence of 0.1 μM Compound A. The Annexin V and propidium iodide staining revealed that there was no enhancement of cell death by the combination treatments in A375 cells. In G361 cells, an enhancement was observed when 0.01 μM of taxol was combined with 0.1 μM Compound A, relative to taxol alone. However, there seems to be no enhancement at higher doses of taxol in G361 cells. We did not observe any enhancement of cisplatin when combined with Compound A in G361 cells.

### 2.5. Induction of Apoptosis by Compound A is Dependent on the Production of Oxidative Stress

**Figure 6:**
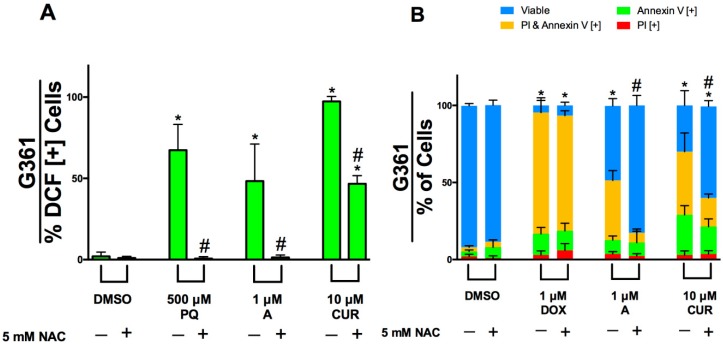
Induction of apoptosis by Compound A is dependent on the production of reactive oxygen species. (**A**) G361 cells were pre-treated with H_2_DCFDA and treated with Compound A and curcumin with or without the antioxidant N-Acetyl-L-cysteine (NAC) for 3 h, as described in the materials and methods section. Production of ROS was evaluated through image-based cytometry with the *y*-axis indicative of the percent of DCF positive cells. *p < 0.05 vs. DCF [+] cells of DMSO control; #*p* < 0.05 vs. DCF [+] cells of groups treated without NAC. (**B**) G361 cells were treated with the Compound A and curcumin with or without NAC for 48 h. Subsequently, cells were stained with Annexin V and PI, as described in the materials and methods. Image based cytometry was utilized to assess apoptosis. The *y*-axis represents the percent of cells positive for Annexin V (green), PI (red), Annexin V and PI (yellow), or negative for both Annexin V and PI (blue). All graphical values are expressed as a mean ± SD from three independent experiments. Doxorubicin (DOX) was used as a positive control. *p < 0.05 vs. DMSO control (comparison of viable cells only); #*p* < 0.05 vs. groups treated without NAC (comparison of viable cells only). PQ = Paraquat, A = Compound A, CUR = Curcumin.

To investigate the role of oxidative stress in apoptosis induction by Compound A we first measured the production of ROS using H_2_DCFDA in G361 cells as indicated in the materials and methods section. Paraquat (PQ) was used as positive control for ROS generation [32]. Overall, Compound A and curcumin exhibited pro-oxidant effects and induced ROS production in G361 cells after 3 h of treatment. This was indicated by a significant increase in the percent of cells positive for DCF relative to the control (Figure 6A). Subsequently, we used the antioxidant N-Acetyl-L-cysteine (NAC) to determine if the observed increase in oxidative stress was essential for the induction of apoptosis by Compound A and curcumin. G361 cells pre-treated with NAC demonstrated a reduction in apoptotic markers indicating that ROS played a critical role in the induction of apoptosis of Compound A and curcumin (Figure 6B).

### 2.6. Compound A and Curcumin Induce Mitochondrial Destabilization in Human Melanoma Cells

**Figure 7:**
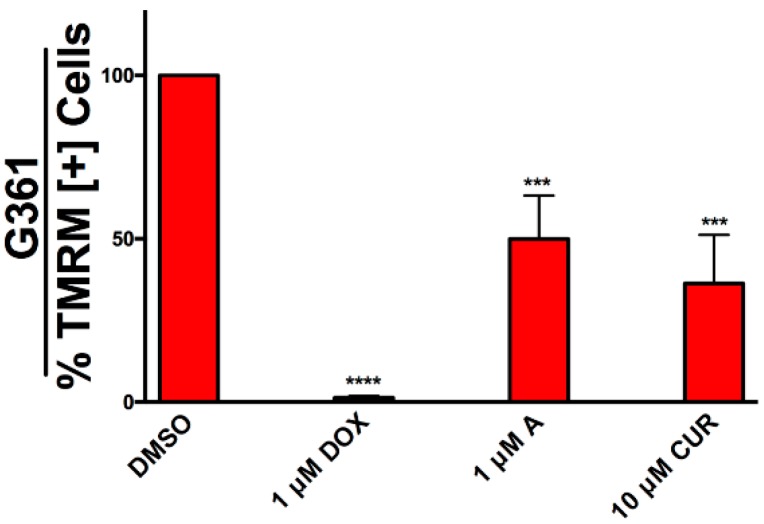
Mitochondrial depolarization in G361 cells undergoing apoptosis. G361 cells were treated with Compound A and curcumin for 48 h followed by incubation with TMRM for 45 min to quantify mitochondrial membrane potential (MMP), as described in the materials and methods section. Results were obtained using image-based cytometry with the *y*-axis representative of percent of cells positive for TMRM expressed as a mean ± SD from three independent experiments. Doxorubicin (DOX) was used as a positive control. A = Compound A, CUR = Curcumin. ***p < 0.001 vs. TMRM [+] cells of DMSO control; ****p < 0.0001 vs. TMRM [+] cells of DMSO control.

Increased production of ROS could lead to the collapse of mitochondrial membrane potential in cells undergoing apoptosis. We investigated if G361 cells undergoing apoptosis induced by Compound A and curcumin exhibited mitochondrial destabilization. G361 cells were treated with Compound A and curcumin for 48 h then stained with tetramethylrhodamine methyl ester (TMRM), an indicator of intact mitochondria, as described in the materials and methods. Compound A and curcumin induced mitochondrial collapse as indicated by a decrease in TMRM positive cells (Figure 7).

### 2.7. Apoptosis Induction by Compound A is Caspase Dependent

**Figure 8:**
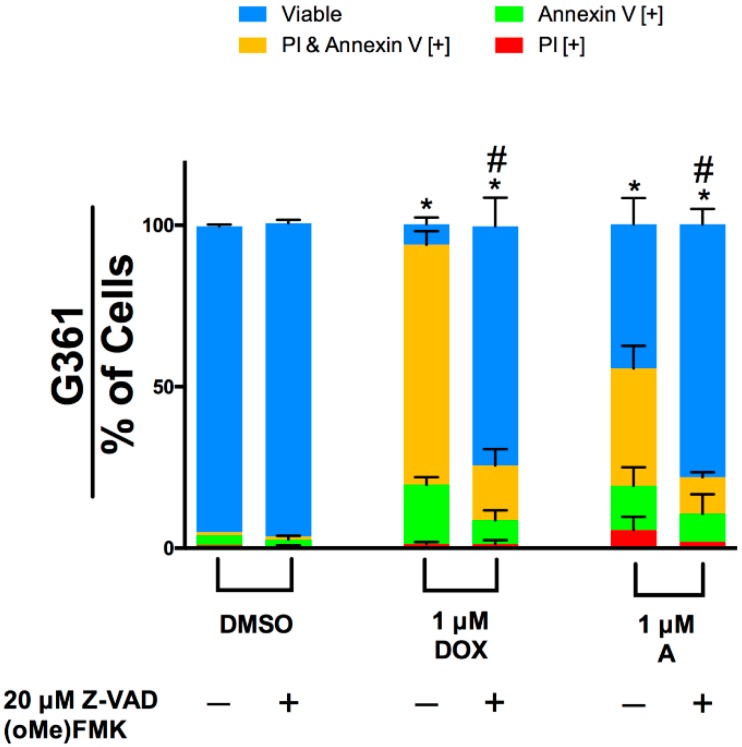
Induction of apoptosis by Compound A is caspase dependent. G361 melanoma cells were pre-treated with or without the caspase inhibitor Z-VAD-FMK for 30 min, followed by treatment with Compound A for 48 h. Cells were stained with Annexin V and propidium iodide to quantify apoptosis. Image based cytometry was utilized to assess apoptosis. The *y*-axis represents the percent of cells positive for Annexin V (green), PI (red), Annexin V and PI (orange), or cells negative for Annexin V and PI (blue). Values are expressed as mean ± SD from three independent experiments. *p < 0.05 vs. DMSO control (comparison of viable cells only); #*p* < 0.05 vs. individual chemotherapeutic treatment (comparison of viable cells only). Doxorubicin (DOX) was used as a positive control. A = Compound A, CUR = Curcumin.

Caspases have been shown to be involved in the initiation and execution phases of apoptosis. To determine if Compound A-induced apoptosis is dependent on caspase activity, G361 cells were pre-treated with or without the broad spectrum caspase inhibitor Z-VAD FMK. Subsequently, the cells were treated with Compound A for 48 h then stained with Annexin V and propidium iodide to characterize apoptosis. A decrease in apoptotic indicators was observed in cells treated with Z-VAD FMK (Figure 8). These results indicate that caspase activity is critical for Compound A induced apoptosis.

## 3. Discussion

In this study, we evaluated the anti-cancer efficacy of synthetic derivatives of curcumin against chemo-resistant human melanoma cells. Out of the ten analogs we screened, Compounds A and I (for structures see Figure 1) were the most effective in reducing cell viability. These analogs induced apoptosis effectively and selectively in melanoma cells. We evaluated the interaction of Compound A with tamoxifen and demonstrated a positive interaction on the apoptosis-inducing activity of Compound A (Figure 4C). We also determined that the induction of apoptosis in cancer cells by Compound A was dependent on the increased production of ROS and caspase activity. Compound A did not interact negatively with taxol and cisplatin in two melanoma cell lines. Overall, we have shown that a novel derivative of natural curcumin, Compound A, targets oxidative vulnerabilities to induce cell death selectively in melanoma cells.

Both melanoma cell lines (A375 and G361) were derived from a primary tumor in malignant melanoma patients. Compounds A and I were found to be very effective at lower doses compared to natural curcumin. Apoptosis is a physiological process important for maintaining homeostasis [30]. Common indicators of apoptosis include DNA condensation or fragmentation, membrane blebbing, and externalization of phosphatidylserine from the inner plasma membrane [30,31]. Analysis of phostatidylserine externalization and permeabilization using the Annexin V binding assay and propidium iodide staining, respectively revealed that these analogs were able to induce apoptosis effectively in A375 cells and at significantly lower concentrations compared to native curcumin. In parallel we tested these compounds on normal human fibroblasts (NHF), a non-transformed noncancerous cell line to investigate the selectivity of Compounds A and I. Importantly, the effective doses of Compounds A and I did not induce apoptosis significantly in NHF cells relative to the DMSO control. Hence, Compounds A and I selectively induce apoptosis in melanoma cells.

Apoptosis can be induced by the extrinsic and intrinsic pathway, which are caused by the activation of a signaling cascade upon binding of an extracellular ligand or a stimulus within the cell, respectively [32]. Both pathways may involve the cleavage and activation of cysteine proteases known as caspases. The intrinsic pathway may be triggered by internal stress, including damage of DNA and oxidative stress [32]. This further causes the mitochondria to lose integrity resulting in the release of apoptogenic factors into the cytoplasm, consequently triggering the induction of apoptosis [33]. Curcumin is a pleiotropic compound with multiple targets and has been characterized to have anti-inflammatory effects [21]. Several researchers have investigated induction of apoptosis targeting mitochondria in cancer cells [34,35,36,37,38,39,40]. Curcumin has been shown to induce apoptosis in a caspase dependent [41,42,43] and independent [44] pathway. We investigated if Compound A induced apoptosis through caspase dependent or independent pathways. Furthermore, the broad caspase inhibitor Z-VAD FMK was able to inhibit Compound A induced apoptosis, indicating a critical role of caspases.

Interestingly, in this study we observed an increased production of ROS following the treatment of melanoma cells with curcumin and Compound A. When curcumin and Compound A were co-treated with the antioxidant NAC, we indeed saw a reduction in ROS generation leading to the inhibition of apoptosis. Thus, Compound A must be targeting oxidative vulnerabilities to induce cell death in melanoma cells. Mitochondrial membrane potential collapse happens at the later stage of apoptosis. Indeed, we observed the collapse of mitochondrial membrane potential (MMP) in melanoma cells following treatment with Compound A and curcumin.

Chemotherapeutic drugs with different targets have the potential to be more effective when combined with a lower chance of resistance [45]. It is integral that we investigate the combined effect of non-toxic, anti-cancer compounds to improve the efficacy of cancer treatments. Tamoxifen is a well-tolerated ER antagonist used for ER positive breast cancer patients and has also been shown to target the mitochondria and induce autophagy [36]. A synergistic induction of apoptosis and autophagy was observed when tamoxifen was combined with an analog of pancratistatin, another mitochondria targeting compound [14]. Likewise, when tamoxifen was combined with curcumin, a synergistic induction of apoptosis and autophagy was observed [29]. We have demonstrated a clear enhancement of Compound A-induced apoptosis by tamoxifen. Importantly, this combination did not exhibit similar toxicity to noncancerous normal human fibroblasts. To our knowledge this is the first time Compound A and tamoxifen have been studied in melanoma cells.

We investigated whether Compound A could be used in conjugation with taxol and cisplatin. Taxol targets the microtubules and causes mitotic arrest [46]. It is used to treat a variety of cancers including melanoma, however is cytotoxic to normal cells [47]. Cisplatin induces apoptosis through DNA damage and is also nonspecific [48]. To determine the drug–drug interactions, we treated A375 and G361 cells with taxol and cisplatin alone or in combination with Compound A. The combination could reveal a positive or negative effect, or no interaction. We did not observe any negative interaction of Compound A with taxol or cisplatin in A375 and G361 cells. However, an enhancement was established at 0.01 μM taxol combined with 0.1 μM Compound A in G361 cells. Overall, Compound A could potentially be combined with taxol and cisplatin, and is safe to use as an adjuvant. Since it is non-toxic compared to other treatments, it could be given over a longer period of time without any adverse side effects.

In conclusion, Compound A is able to induce apoptosis effectively in drug resistant melanoma cells with ten times more efficacy than natural curcumin by targeting oxidative vulnerabilities. The anti-cancer activity of Compound A is enhanced by tamoxifen and the combination treatment was well tolerated by normal cells. Compound A did not interact negatively with taxol and cisplatin. In fact, a positive interaction was observed in G361 cells treated with taxol and Compound A. Compound A could potentially be developed as a safe and effective treatment alone or in combination with tamoxifen. Future work may focus on elucidating the mechanism and targets of Compound A induced cell death, and in vivo models of melanoma should be tested to determine efficacy and stability.

## 4. Materials and Methods

### 4.1. Chemical Synthesis of Curcumin Analogs

All chemical reagents were obtained from Sigma–Aldrich, Fluka, and Aladdin (Beijing, China). Synthesis of Compounds A-J has been described previously [24].

### 4.2. Cell Culture

Malignant melanoma cell line A375 (ATCC, Cat. No. CRL-1619, Manassas, VA, USA) was cultured in RPMI-1640 medium (Sigma–Aldrich Canada, Mississauga, ON, Canada) supplemented with 10% (v/v) fetal bovine serum (FBS) standard (Thermo Scientific, Waltham, MA, USA) and 40 μg/mL gentamicin (Gibco BRL, VWR, Mississauga, ON, Canada).

Malignant melanoma cell line G361 (ATCC, Cat. No. CRL-1424, Manassas, VA, USA) was cultured in McCoy’s 5A Medium supplemented with 2 mM L-glutamine, 10% (v/v) FBS (Thermo Scientific, Waltham, MA, USA), and 40 μg/mL gentamicin (Gibco BRL, VWR, Mississauga, ON, Canada).

The noncancerous cell line, normal human skin fibroblast cells (NHF; Coriell Institute for Medical120 Research, Cat. No. AG09309, Camden, NJ, USA) were cultured in Dulbecco’s Modified Eagle’s Medium (DMEM; ATCC^®^ 30-2002TM) supplemented with 10% (v/v) fetal bovine serum (FBS) and 40 μg/mL gentamicin (Catalog No. 15710-064, Gibco BRL, VWR, Mississauga, ON, CA).

All cultured cells were maintained in an incubator at 37 °C, 5% CO_2_, and 95% humidity. The cells were passaged for less than six months.

### 4.3. Chemicals and Cell Treatment

Human melanoma cells and NHF cells were grown to roughly 70% confluence and then treated with curcumin (CUR; Sigma-Aldrich Canada, Cat. No. 8511, Mississauga, ON, Canada), Compound A and other curcumin analogs (e.g., Compound I) all dissolved in dimethylsulfoxide (DMSO) to make the stock solutions. In parallel, these cells were treated with taxol (Sigma–Aldrich Canada, Cat. No. T7402, Mississauga, ON, Canada), cisplatin (Sigma–Aldrich Canada, Mississauga, ON, Canada), doxorubicin (DOX; Sigma–Aldrich Canada, Cat. No. D1515, Mississauga, ON, Canada), and Z-VAD-FMK (Sigma–Aldrich Canada, Cat. No. V116, Mississauga, ON, Canada). These agents were also dissolved in dimethylsulfoxide (DMSO) to make the stock solutions. *N*-Acetyl-L-cysteine (NAC; Sigma–Aldrich Canada, Cat. No. A7250) and paraquat (PQ; Sigma–Aldrich Canada, Cat. No.856177, Mississauga, ON, Canada) were dissolved in double distilled water.

### 4.4. WST-1 Assay for Cell Viability

A WST-1 colorimetric assay was used to quantify cell viability of cells treated with curcumin and its analogs (compound A-J), following a previously published protocol [15]. Briefly, A375 and NHF cells were seeded in ninety-six well clear bottom tissue culture plates then treated with the indicated compounds for 48 h. The cells were then incubated with the WST-1 reagent (Roche Applied Sciences, Indianapolis, IN, USA) for 4 h at 37 °C, 5% CO2, and 95% humidity. The WST-1 reagent is cleaved by viable cells to formazan through its enzymes. Absorbance was read at 450 nm on a Wallac Victor3 1420 Multilabel Counter (PerkinElmer, Waltham, MA, USA) to determine cell viability.

### 4.5. Analysis of Cell Death

Annexin V binding assay and propidium iodide staining were used to determine early apoptosis and cell permeabilization (found in late apoptotic cells), respectively as described previously [15]. Briefly, melanoma and NHF cells were seeded in six-well plates and after 24 h they were treated with different compounds at various concentrations for 48 h. Cells were washed with phosphate buffer saline (PBS), and then Annexin V Binding buffer (10 mM HEPES, 140 mM NaCl, 2.5 mM CaCl2, pH 7.4) was added, followed by the Annexin V AlexaFluor-488 dye, which fluoresces a green color (1:20; Life Technologies Inc., Cat. No. A13201, Burlington, ON, Canada). Afterwards, 0.01 mg/mL of propidium iodide dye (red fluorescent color) was added (Life Technologies Inc, Cat. No. P3566, Burlington, ON, Canada). Afterwards, the cells were incubated for 15 min at 37 degrees Celsius, 5% CO2, and 95% humidity in the absence of light. A Tali Image-Based Cytometer (Life Technologies Inc., Cat. No. T10796, Burlington, ON, Canada) was used to quantify the percentage of cells stained green (early apoptotic), green and red (late apoptotic), and red (necrotic). Cells from 18 random fields were used to analyze the green (ex. 458 nm; em. 525/20 nm) and red (ex. 530 nm; em. 585 nm) channels. In order to visualize cellular death and apoptosis, a similar protocol was carried out. Cells were stained with propidium iodide and Hoechst 3342 (Molecular Probes, Eugene, OR, USA) at 10 μM during the 15 min incubation. Fluorescent and Brightfield micrographs were taken at 400× magnification using a Leica DMI6000 fluorescent microscope with the LAS AF6000 software.

### 4.6. Tetramethylrhodamine Methyl Ester Staining

Tetramethylrhodamine methyl ester (TMRM; Thermo Fisher Scientific) stain was used to determine mitochondrial membrane potential (MMP) as outlined in a previously published protocol [24]. G361 cells were cultured and then treated with doxorubicin, Compound A, and curcumin for 48 h. Subsequently, the cells were incubated with 100 nM TMRM for 45 min at 37 °C. Cells were washed with PBS, and then a Tali Image-Based Cytometer (Life Technologies Inc., Cat. No. T10796, Burlington, ON, Canada) was then used to quantify the percentage of red stain from TMRM (stable mitochondrial membrane potential). Cells from 18 random fields were used to analyze the red (ex. 530 nm; em. 585 nm) channel.

### 4.7. Quantification of Reactive Oxygen Species

The molecule 2′,7′-dicholorofluorescin diacetate (H_2_DCFDA) was used to determine whole-cell reactive oxygen species (ROS) generation as outlined in a previously published protocol [24]. The molecule is deacetylated by esterases and oxidized by ROS into a fluorescent green 2′,7′-dicholorofluorescein (DCF; ex. 495 nm; em. 529 nm) once inside the cell. G361 cells were pretreated with a final concentration of 20 μM H_2_DCFDA (D6883; Sigma-Aldrich) and then incubated for 30 min at 37 °C and 5% CO_2_ in the absence of light. Afterwards, the cells were treated with various drug concentrations for 3 h. The cells were centrifuged at 3000 rpm for 5 min and then suspended in PBS. A Tali Image-Based Cytometer (Life Technologies Inc., Cat. No. T10796, Burlington, ON, Canada) was then used to quantify the percentage of green fluorescent cells from the presence of DCF-positive stain. Cells from 18 random fields were used to analyze the green (ex. 458 nm; em. 525/20 nm) channel. In order to determine the mechanism of action of Compound A and curcumin, G361 cells were pretreated with or without *N*-Acetyl-L-cysteine (NAC; final concentration 5 mM; Sigma–Aldrich Canada, Cat. No. A7250) and incubated for 30 min. NAC acts as an antioxidant which inhibits the presence of ROS in the cell. H_2_DCFDA was utilized to determine ROS generation as mentioned above. Subsequently, the cells were treated with DMSO, paraquat, Compound A, and curcumin. The cells were then read for the presence of green fluorescence at a time point of 3 h following incubation using a Tali Image-Based Cytometer (Life Technologies Inc., Cat. No. T10796, Burlington, ON, Canada).

### 4.8. Caspase Inhibition

Cell treatment with or without general caspase inhibitor Z-VAD-FMK was used to determine if the apoptotic mechanism of Compound A was caspase dependent or independent. Cells were pretreated with 4 μL Z-VAD-FMK (final concentration 20 μM) for 30 min at 37 °C at 5% CO_2_. Cells were then treated with DMSO, doxorubicin (DOX), and Compound A. After incubation for 48 h, cells were collected, washed with phosphate buffer saline (PBS), and apoptosis was quantified using the Annexin V Binding Assay protocol as previously described.

### 4.9. Statistical Analysis

Statistical analysis was conducted by the GraphPad Prism 6 software. Significance was considered when the *p*-value was less than 0.05. The type of statistical analysis depended on the experimental variable. Single variable measurements, including quantification of mitochondrial membrane potential, and whole cell ROS, were analyzed by the one-way ANOVA (nonparametric) and the mean of each sample was compared to the mean of the negative control (DMSO) unless otherwise specified. Multi-variable experiments such as the quantification of early and late apoptosis, were analyzed by two-way ANOVA (nonparametric) and the sample of each mean was compared to the mean of the negative control (DMSO) unless otherwise specified.
